# Occurrence and outcome of firework-related ocular injuries in Switzerland: A descriptive retrospective study

**DOI:** 10.1186/s12886-022-02513-9

**Published:** 2022-07-07

**Authors:** Ferhat Turgut, Alexandra Bograd, Brida Jeltsch, Adrian Weber, Petra Schwarzer, Iulia M Ciotu, Joao Amaral, Marcel N Menke, François Thommen, Tamer Tandogan, Christoph Tappeiner

**Affiliations:** 1 Department of Ophthalmology, Inselspital, Bern University Hospital, University of Bern, Bern, Switzerland; 2 Department of Ophthalmology, City Hospital Triemli, Zurich, Switzerland; 3 Department of Ophthalmology, University Hospital of Zurich, Zürich, Switzerland; 4 Department of Ophthalmology, Lucerne Cantonal Hospital, Lucerne, Switzerland; 5 Department of Ophthalmology, Pallas Klinik Olten, Louis Giroud-Strasse 20, 4600 Olten, Switzerland; 6 Department of Ophthalmology, University Hospitals of Geneva, Geneva, Switzerland; 7 Department of Ophthalmology, Kantonsspital Aarau, Aarau, Switzerland; 8 Department of Ophthalmology, University of Lausanne, Hôpital Ophtalmique Jules-Gonin, Lausanne, Switzerland; 9 Department of Ophthalmology, University Hospital Essen, University Duisburg-Essen, Essen, Germany; 10 University of Bern, Bern, Switzerland

**Keywords:** Firework, Ocular trauma, Occurrence, Outcome, Surgery, Switzerland

## Abstract

**Background:**

Firework-related ocular injuries (FWROI) are a major cause of preventable visual impairment. This study aimed to analyze the occurrence and outcome of FWROI in Switzerland.

**Methods:**

This retrospective multicenter study included patients with FWROI from seven centers in Switzerland from January 2009 to August 2020. Demographic information, type of injuries, medical and surgical treatments, the best corrected visual acuity (BCVA) at baseline and end of follow-up, occurrence and type of secondary complications, and duration of hospitalization were analyzed.

**Results:**

A total of 105 patients (119 eyes) with a mean age of 27.1 ± 15.9 years were included in the study (71.4% male patients; 29.5% underage). Most injuries occurred around New Year’s Eve (32.4%) and the Swiss national holiday on 1 August (60.9%). The most common anterior segment findings were conjunctival or corneal foreign bodies (58%), whereas Berlin’s edema was the most common posterior segment finding (11.4%). Globe ruptures were found in four patients. The mean BCVA in all patients at first presentation was 0.4 ± 0.8 logMAR and improved to 0.3 ± 0.8 logMAR at last follow-up. A primary surgical intervention was performed in 48 eyes (40.3%). Hospitalization directly after the trauma was necessary for 18 patients for a mean of 5.8 ± 4.1 days, and a total of 4.9 ± 7.6 follow-up visits were needed.

**Conclusion:**

This study provides the first data on FWROI in Switzerland, which are helpful for further preventive and educational programs and comparisons with other countries.

**Supplementary Information:**

The online version contains supplementary material available at 10.1186/s12886-022-02513-9.

## Background

Fireworks are used for celebrating all kinds of special occasions worldwide such as New Year’s Eve or national holidays. The Fireworks Annual Report 2019 by the United States Consumer Product Safety Commission estimated that in 2019 a total of 10,000 firework-related injuries – of which about 15% involved ocular trauma – were treated in U.S hospital emergency departments [1]. Similar data regarding the prevalence of ocular involvement in firework accidents have also been published for other countries. Wang et al. reported that the most frequently injured body parts due to fireworks in Beijing (China) were hands and fingers (32%), whereas in 11.4% of cases the eyes were also involved [2]. In a systematic review on firework-related ocular injuries (FWROI), ocular trauma was found in 21.8% (range 16–45%) of firework victims [3]. The unlawful or inappropriate use of fireworks (e.g., not following the instructions of the manufacturer, or using fireworks under the influence of alcohol or drugs) may lead to mild superficial ocular injuries but also to severe trauma involving the whole globe and surrounding tissues [3, 4]. Depending on the trauma mechanism, ophthalmologists are confronted with complex injuries such as globe penetration, perforation, or even rupture for which emergency surgeries are required. Such injuries often lead to secondary complications that require further medical treatment and eventually also to repeated ocular surgeries to prevent irreversible vision loss [5]. FWROI may also have a significant impact on quality of life and potentially lead to a temporary or even a long-term inability to work [5]. As up to 35% of patients with FWROI are younger than 18 years [4], a potential long-term disability could have both critical economic consequences and a relevant impact on the public health system. To the best of our knowledge, only limited data about firework-related injuries are available for Switzerland. A smaller retrospective study about general firework-related injuries from 2013 to 2019 at the University Emergency Department of the Inselspital in Bern [6] and another retrospective study concerning FWROI patients presenting to the Department of Ophthalmology of the University Hospital in Zurich from 2011 to 2016 have been published recently [7]. While both studies provided valuable information about firework-related injuries, the data were limited due to the monocentric study designs and the short observation period. This multicenter study thus aimed to analyze the occurrence and the outcome of FWROI in Switzerland over a longer period and to include all major hospitals in the assessment.

## Methods

This multicenter retrospective study included all patients who presented to the emergency departments and departments of ophthalmology of almost all major hospitals in Switzerland due to an FWROI from 1 January 2009 to 31 August 2020. The seven participating centers were Inselspital Bern, Universitätsspital Zürich, Luzerner Kantonsspital, Pallas Kliniken, Hôpitaux Universitaires de Genève, Kantonsspital Aarau, and Hôpital Jules-Gonin Lausanne. No data from the Luzerner Kantonsspital was available for the period from January 2009 to May 2014. Ethical approval was obtained from all responsible ethical boards. The study was conducted in accordance with the declaration of Helsinki.

The medical history of all patients fulfilling the inclusion criteria was extracted at all study-centers by a medical chart full-text search, excluding the data of patients who had refused the general consent. For all patients, a case report form (Suppl. Table 1) was filled out by the local investigators. In the first part, demographic data were queried followed by information about accident details such as the role of the patient (bystander vs. operator of the firework) and the injured eye/s. The best corrected visual acuity (BCVA), intraocular pressure (IOP), and clinical findings at first presentation (baseline) and at follow-up visits were provided. Furthermore, information about medical and surgical treatments and ocular complications was collected. To determine the socio-economic impact, data about primary and secondary hospitalization, duration of sick leave, and the number of ophthalmological consultations were assessed.

Descriptive statistics were performed. The primary endpoint was the occurrence of FWROI. To analyze the outcome of FWROI, the following parameters were investigated as secondary endpoints: type of injuries, primary and secondary medical and surgical treatments, the outcome of BCVA, occurrence and type of secondary complications, duration of hospitalization, number of necessary ophthalmological visits, and duration of sick leave. Due to the descriptive nature of the study, no statistical comparisons were performed, and only descriptive data are presented. Continuous data are presented with means ± standard deviation. Microsoft Office 2020 was used to create the graphs.

## Results

**Fig. 1:**
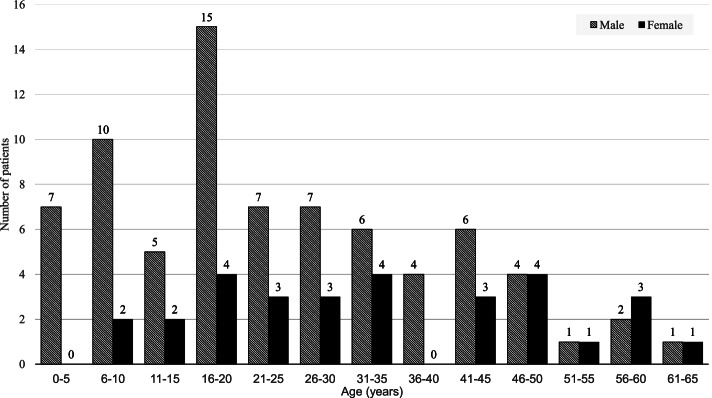
Age and sex distribution of patients with FWROI (*n* = 105 patients)

A total of 105 patients (119 eyes) with FWROI presenting to participating clinics between 1 January 2009 and 31 August 2020 were included in this retrospective analysis (mean follow-up time of 15.4 ± 30.8 months; range 0.03–132.5 months). The mean age of the patients was 27.1 ± 15.9 years (range 2–65) at first presentation, of which 31 patients (29.5%) were underage (Fig. 1). The three youngest patients were two years of age and presented with foreign bodies under the eyelids and corneal epithelial defects. The sex distribution, patients’ role during the firework accident, and injured eye/s are presented in Table 1. Most patients were male (71.4%). A total of 15 patients (14.3%) acted as firework operators during the incident, whereas 54 (51.4%) were bystanders, and the role of 34.3% of patients is unknown. A total of 13 out of 15 (86.7%) injured firework operators were male. In 48 patients (45.7%) the right eye and in 43 patients (41%) the left eye was injured, whereas in 14 patients (13.3%) both eyes were involved. All patients with bilateral FWROI were male and of these, four were firework operators, five bystanders, and five with an unknown role.

**Table 1:** Demographics of patients (*n* = 105) with FWROI between January 2009 and August 2020 in Switzerland

	**Patients, n (%)**	**Male, n (%)**	**Female, n (%)**
**Sex**		75 (71.4)	30 (28.6)
**Role during the fireworks display**			
Bystander/spectator	54 (51.4)	35 (64.8)	19 (35.2)
Operator/handling/lighting	15 (14.3)	13 (86.7)	2 (13.3)
Unknown	36 (34.3)	27 (75)	9 (25)
**Injured eye**			
Right eye	48 (45.7)	28 (58.3)	20 (41.7)
Left eye	43 (41)	33 (76.7)	10 (23.3)
Both eyes	14 (13.3)	14 (100)	0 (0)

**Fig. 2:**
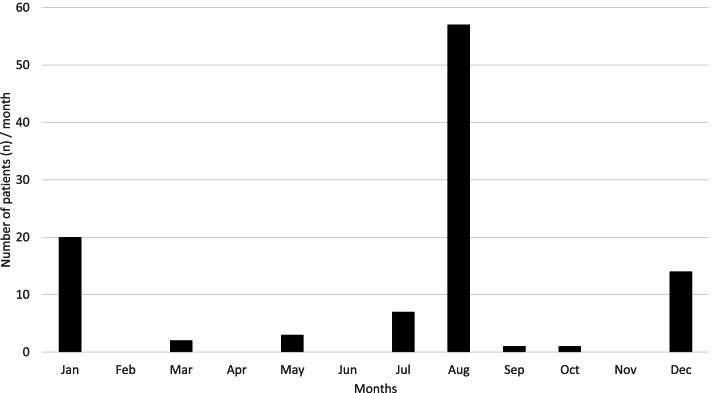
The annual distribution of FWROI from January 2009 to August 2020. Two spikes can be observed: 34 patients (32.4%) presented around New Year’s Eve and 64 (60.9%) on the Swiss national holiday on 1 August, respectively

**Fig. 3:**
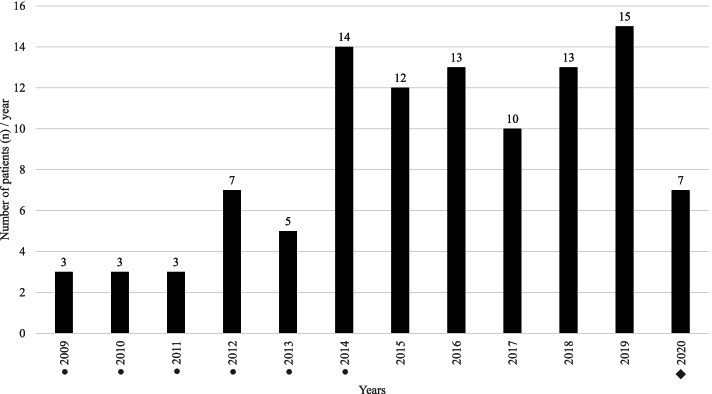
The occurrence of firework-related ocular injuries per year (mean 8.8 ± 4.6 patients with FWROI per year). ● No systematic search to identify study subjects was feasible for emergency patients of the Luzerner Kantonsspital for the period of January 2009 to June 2014. ◆ Patients with FWROI between September and December 2020 were not included due to the end of the study period on 31 August 2020

The annual distribution of FWROI reveals two peaks (Fig. 2): 34 patients (32.4%) presented around New Year’s Eve and 64 (60.9%) around the Swiss national holiday on 1 August, which is usually celebrated with fireworks. Only seven injuries (6.7%) were not related to these public celebrations. This corresponds to a mean of 8.8 ± 4.6 patients with FWROI per year in the study period (Fig. 3).

**Fig. 4:**
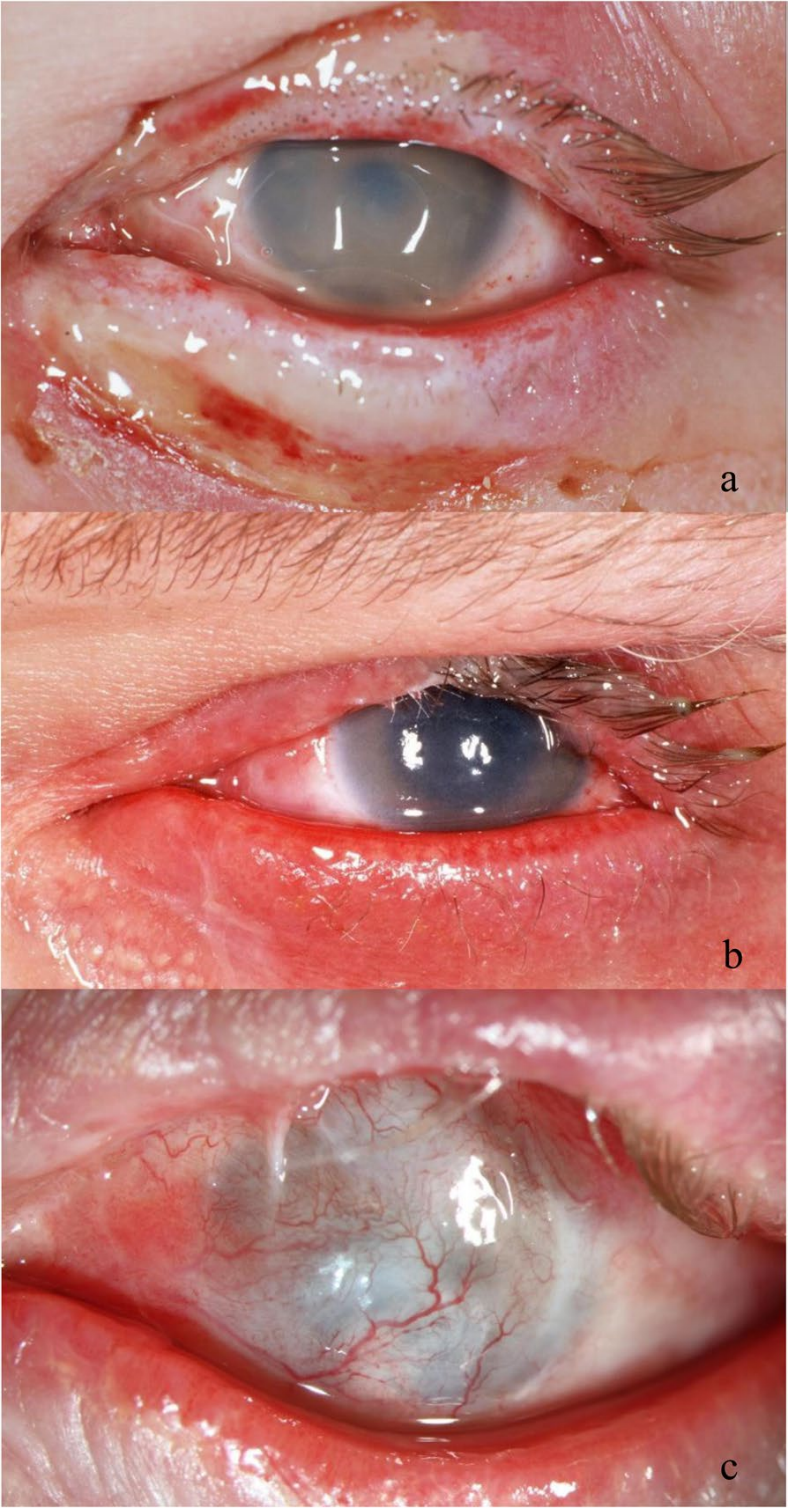
Exemplary photographs of a 22-year-old patient with a firework-lighting injury of his left eye: (a) The patient presented with an extensive burn of the eyelid, conjunctiva, and cornea and a total corneal epithelial defect. The visual acuity was light perception. (b) One month later, corneal opacification, limbal stem cells deficiency, and visual acuity of hand motion were observed. (c) During further follow-up and following multiple ocular surgeries, the eye developed a complete conjunctivalization and vascularization of the cornea and ocular phthisis

The ocular and adnexal injuries at first presentation are shown in Table 2. A globe rupture was diagnosed in four patients, and one other patient had an orbital fracture. The most common anterior segment findings were conjunctival or corneal foreign bodies in 69 eyes (58%) and corneal epithelial defects in 61 eyes (51.3%) (Fig. 4). Involvement of the eyelids was observed in 27 patients (25.7%), most frequently in the form of eyelid burns (17.6%). The most common posterior segment finding was Berlin’s edema in 12 patients (10.1%).

**Table 2:** Clinical findings at first presentation of 105 patients (*n* = 119 eyes) with FWROI

**Clinical findings**	**Eyes, n (%)**
Corneal epithelial defect	61 (51.3)
Conjunctival foreign body	46 (38.7)
Corneal foreign body	23 (19.3)
Traumatic iritis	23 (19.3)
Lid burn	21 (17.6)
Charred eyelashes	18 (15.1)
Hyposphagma	16 (13.4)
Chemosis	14 (11.8)
Conjunctival burn	13 (10.9)
Traumatic mydriasis	13 (10.9)
Berlin’s edema	12 (10.1)
Conjunctival laceration	12 (10.1)
Hyphemia	12 (10.1)
Corneal perforation	7 (5.9)
Vitreous hemorrhage	7 (5.9)
Corneal burn	6 (5.0)
Retinal hemorrhage	6 (5)
Hematoma periocular	5 (4.2)
Eyelid laceration	5 (4.2)
Scleral laceration	4 (3.4)
Corneoscleral laceration	4 (3.4)
Globe rupture	4 (3.4)
Traumatic cataract	3 (2.5)
Iridodialysis	2 (1.7)
Aniridia	2 (1.7)
Intravitreal foreign body	2 (1.7)
Choroidal rupture	2 (1.7)
Others^a^	6 (6.7)

^a^Other clinical findings which were each found in one eye only, were traumatic ptosis**,** corneal laceration**,** intracameral foreign body, angle recession**,** lens dislocation, retinal detachment**,** traumatic retinal break, and orbital fracture

**Fig. 5:**
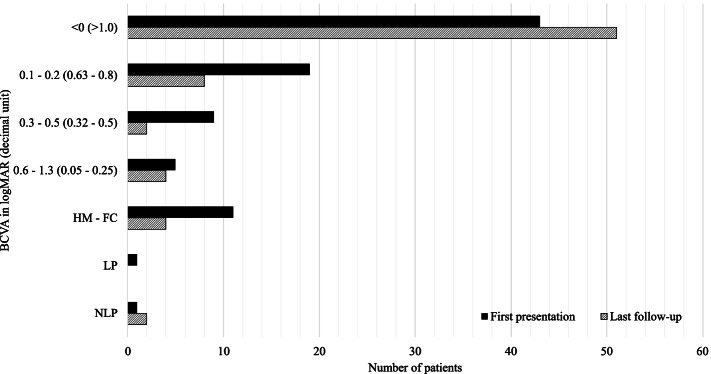
Best corrected visual acuity (BCVA) at first presentation after injury (available for 89 eyes) and at last follow up (available for 71 eyes) is depicted for different decimal unit ranges. NLP = no light perception, LP = light perception, HM = hand motion, FC = finger counting

BCVA data at first presentation were available for 89 patients with a mean of 0.4 ± 0.8 logMAR and ranging from no light perception (*n* = 1), light perception (*n* = 1), hand motion (*n* = 6), and finger counting (*n* = 6) up to 20/16 (decimal unit of 1.6) (Fig. 5). The final follow-up mean BCVA improved to 0.3 ± 0.7 logMAR. Information on the IOP at first presentation was available for 85 eyes and revealed a mean of 13.8 ± 5.2 mmHg (range 0–32), whereas at the last follow-up visit it was 14.4 ± 3.9 mmHg (range 7–32).

A primary surgical intervention was performed in 48 eyes (40.3%) of 39 patients (37.1%), including minor slit lamp procedures (Table 3). The most frequently performed procedure was the removal of conjunctival or corneal foreign bodies (45 eyes, 37.8%), which was mainly performed at the slit lamp. Major primary surgeries such as globe explorations or pars plana vitrectomies were performed in eight (6.7%) and two (1.7%) eyes, respectively.

**Table 3:** Primary surgical interventions

Primary surgical intervention	Eyes, n (%)
Extraction of conjunctival or corneal foreign bodies	45 (37.8)
Corneal/corneoscleral suture repair	9 (7.6)
Globe exploration	8 (6.7)
Conjunctival suture repair	7 (5.9)
Scleral suture repair	5 (4.2)
Eyelid suture repair	4 (3.4)
Anterior chamber washout	3 (2.5)
Vitrectomy	2 (1.7)
Corneal epithelium abrasion	2 (1.7)
Amniotic membrane transplantation	2 (1.7)
Lensectomy	1 (0.8)
Extraction of intraocular foreign bodies	1 (0.8)

Primary surgical interventions, including minor slit lamp procedures, were performed in 48 eyes (*n* = 39 patients) out of 119 eyes (*n* = 105 patients) with FWROI

Due to secondary complications, a later ocular surgery had to be performed in 11 eyes after a mean of 7.6 ± 10.0 months (range 0.1–33.0) following the FWROI: seven vitrectomies (four of them with silicon oil tamponade), two penetrating keratoplasties, two lensectomies, two secondary intraocular lens implantations, and two eviscerations. A total of four patients with an age of 16, 26, 37, and 38 years had a globe rupture. In one of these patients, an evisceration had to be performed two years after the injury and following several other ocular surgeries such as two keratoplasties and a vitrectomy. Of these four patients with globe ruptures at first presentation, BCVA was available for two of them: one revealed hand motion and the other one light perception. At the last follow-up, the BCVA in these four patients was no light perception, hand motion, finger counting, and 20/2,000 (decimal unit of 0.1), respectively.

Topical treatment was necessary for all 105 patients (119 eyes), whereas additional systemic treatment was given in 22 patients (Table 4). The most frequently used treatments were topical antibiotics (73.9%), lubricating eye drops (53.8%), and topical corticosteroids (52.9%). Due to foreign bodies such as ash or other solid particles from blasting fireworks, 23 eyes (19.3%) had to be irrigated. Topical IOP lowering agents were used in six eyes (five patients) and systemic IOP lowering medication was prescribed in one patient.

**Table 4:** Primary medical management directly after the occurrence of FWROI in Switzerland

	Eyes, n (%)	
**Topical drugs**		
Topical antibiotics	88 (73.9)	
Lubricating eye drops	64 (53.8)	
Topical corticosteroids	63 (52.9)	
IOP lowering agents	6 (5)	
Cycloplegics	4 (3.4)	
Topical nonsteroidal anti-inflammatory drugs	3 (2.5)	
**Irrigation**	23 (19.3)	
	**Patients, n (%)**	
**Systemic drugs**		
Systemic nonsteroidal anti-inflammatory drugs	16 (15.4)	
Systemic antibiotics	10 (9.6)	
IOP lowering medication	1 (1)	
**Others**		
Tetanus prophylaxis	3 (2.9)	

Hospitalization directly after the trauma was necessary for 18 patients (mean age of 28.4 ± 14.4 years; range 4–65) for a mean of 5.8 ± 4.1 days. All patients were male and required surgery. Five patients had to be hospitalized for a mean of 6.2 ± 5.6 days at a later time-point due to secondary complications. Sick leave had to be issued for 23 patients: these patients were unable to attend work or school for a mean of 64.2 ± 130.1 days (range 1–540 days). The patients needed a mean of 4.9 ± 7.6 ophthalmological consultations (range 1–40 visits) due to their FWROI.

## Discussion

Around the world, fireworks are used on special occasions and especially also on national holidays: New Year’s Eve [8–10], at the Spring festival in China [2], at Diwali in India [11, 12], and at the Persian Eve Festival [13, 14]. In Switzerland, fireworks displays are also especially common on New Year’s Eve on 31 December and around the period of the Swiss national holiday on 1 August. This may lead to variable injury patterns during these seasons, which we have also confirmed in our study with 93.3% of all injuries occurring during these times (32.4% and 60.9% around New Year’s Eve and the Swiss national holiday, respectively). Similar results have been reported for the United States, where up to 85% of firework-related injuries occurred in association with celebrations of Independence Day and New Year’s Eve [10].

The demographic data we gathered is similar to the reported findings from other countries. In our study, about 70% of the patients were male, which is similar to the 75% (range 66–95%) that were identified in the metanalysis by Wisse et al. [3]. Furthermore, 62% of our patients were under the age of 30 years, which compares well to the 67% reported by Wisse et al. [3]. The rate of bystander involvement varied in other countries from 39% in Saudi Arabia [15], 40–53% in the Netherlands [5, 16], 56% in Germany [17], up to 64% in the United Kingdom [18], whereas in our study 51% of those injured were bystanders. However, missing information concerning the role played in the firework accident for 34% of our patients might represent a potential bias.

In our study, three different injury patterns were observed, that either occurred in isolation or in combination with each other: (a) conjunctival or corneal foreign bodies, (b) burn of the eyelid, conjunctiva or cornea, and/or (c) a blast injury leading to laceration or even globe rupture. Furthermore, the majority of FWROI were mild and full remission was achieved. Patients most suffered from an isolated superficial corneal lesion (51%). Similar results have also been reported in studies from countries such as Sweden, Germany, and India with corneal lesions in 54%, 42%, and even 80% of cases, respectively [12, 17, 19]. Regarding the rate of severe trauma such as globe rupture, the occurrence in our study with 3% was much lower than in other studies (mean of 15%, range 9–23%) [3, 4], leading to a two times lower need for enucleation/evisceration in our cohort. Significantly higher enucleation rates in patients with FWROI were reported for other countries (e.g., 18% enucleation rate in the United States for patients with FWROI) [20]. However, discrepancies might also be explained by under-reporting less severe trauma in certain registries/studies and a potential selection bias. National trauma registries that also document FWROI have been introduced in several countries, e.g., North America (US Consumer Product Safety Commission) [1], United Kingdom (Consumer Safety Unit of the Department of Trade and Industry), or the Netherlands (the Consumer and Safety Foundation) [21]. However, for many other countries, limited or no data about FWROI are available. To the best of our knowledge, there is no central Swiss registry that allows to extract detailed and complete data of firework-related ocular injuries. Such a database would be highly desirable for a reliable epidemiological overview of the situation in Switzerland. The benefits of trauma registries for improved patient care and for developing prevention strategies have been widely researched [22, 23].

FWROI can have a severe negative impact on the quality of life which might be preventable if specific measures, such as a firework ban or education on handling explosives, are implemented by governments. Experts emphasize the positive influence of a firework ban on the occurrence of firework-related injuries. For example, the lifting of a firework ban in Ireland in 1996 that permitted the sale and use of pyrotechnic articles for private displays led to a significant increase in ocular firework injuries [24]. While six patients were admitted to the Royal Victoria Hospital Eye Department from 1990 to 1995, the number of patients increased to 17 from 1996 to 2001. Therefore, a ban on fireworks was re-introduced in 2002, although further follow-up of FWROI in Ireland is not available as far as we know. In the United States, the Michigan State Legislature passed the Fireworks Safety Act in 2011 and thereby legalized the sale of consumer fireworks, such as fireworks that leave the ground, whereas previously only on-ground fireworks were legal. After expanding the possibility to purchase fireworks of different categories, the incidence rate of firework-related injuries increased from 14.3/100,000 to 21/100,000 [25]. On the other hand, the legal purchase of fireworks and governmental regulation through standardization may prevent the use of highly dangerous self-made or illegally purchased devices, with a high risk of severe trauma due to premature or delayed blast [26]. The World Health Organization recommended government regulations of the manufacture and use of fireworks [27]. In Switzerland the use of fireworks is regulated in the Ordinance on Explosives [28]. Fireworks are subdivided into categories F1 to F4 according to their level of danger (F1 low hazard, F4 major hazard). Category F1-F3 fireworks can be purchased in public shops and are subjected to age restrictions (F1, F2 and F3 purchase possible above 12, 16 and 18 years of age, respectively). Fireworks in category F4 are reserved for commercial usage and may only be purchased and handled by specialists with pyrotechnical knowledge. Furthermore, special fireworks such as “lady-crackers” longer than 22 mm and/or with a diameter of more than 3 mm are prohibited. Firecrackers with a horizontal explosive mode of action are not allowed in Switzerland.

Not only the limitation of firework sales but also preventive measures such as educational campaigns might help to reduce firework-related accidents [29, 30]. The Swiss Advisory Center for Fire Protection (Beratungsstelle für Brandverhütung), the Swiss Council for Accident Prevention (Beratungsstelle für Unfallverhütung) and the Swiss National Accident Insurance Fund (Schweizerische Unfallversicherungsanstalt) have published recommendations for the safe handling of fireworks [31–33].

The relevant number of children affected by FWROI is concerning and may be reduced by raising public awareness [11, 12, 34–36]. Different studies highlighted the role played by a lack of supervision in children with firework injuries (up to 80% according to Bagri et al.) [11, 20]. The three youngest patients in our study were only two years old and were also unsupervised during the firework accident. From this, it is clear that highlighting the importance of parental supervision in educational campaigns is necessary.

There is a relevant economical aspect of firework sales: in 2017, a total of 885 million US dollars was generated with consumer sales of fireworks in the United States showing an increase of 41% compared to 2008 [37]. On the other hand, the number of firework-related injuries increased accordingly from 2,576 in 2008 to 5,101 in 2017 [37]. This also results in an increased financial burden to health systems due to the need for medical visits, hospitalizations, and surgeries. According to van Yperen et al., the mean total health costs for patients with firework-related injuries were 6,320 € per patient in the Netherlands [38]. Additionally, the high level of sick leave, rehabilitation measures, and temporary or persisting invalidity have a relevant socioeconomic impact and burden the social welfare system.

This study has potential limitations such as the retrospective character with missing or incomplete data. No systematic search to identify study subjects was feasible for emergency patients of the Luzerner Kantonsspital for the period from January 2009 to June 2014 (paper medical charts). Furthermore, two out of eight hospitals of maximum care (i.e., A-hospitals) did not participate in this study. As patients with minor ocular injuries might also present to local ophthalmologists or to an emergency department without ophthalmological care and not to the major emergency and ophthalmology centers in Switzerland that participated in this study, a certain selection bias has to be assumed. Consequently, the results of our study cannot be considered as epidemiological data but, nevertheless, for the first time provide a good overview of the situation in Switzerland.

## Conclusion

This study provides the first data about the occurrence and characteristics of FWROI for almost all major ophthalmology and emergency departments in Switzerland. This information is pivotal for further preventive and educational programs, for comparisons with other countries, and most importantly, for advocacy at the local and international levels. The results may also help to assess the need for potential legislative measures, such as considering the limitation of public sales of hazardous fireworks in Switzerland.

## Supplementary Information

**Additional file 1: **Case report form used by all participating centers in this retrospective study.

## Data Availability

The datasets generated during and/or analyzed during the current study are available from the corresponding author on reasonable request.

## Abbreviations

FWROI: Firework-related ocular injuries
BCVA: Best corrected visual acuity
IOP: Intraocular pressure
NLP: No light perception
LP: Light perception
HM: Hand motion
FC: Finger counting
