# Supplementary material for: Occurrence and outcome of firework-related ocular injuries in Switzerland: A descriptive retrospective study
Source: BMC Ophthalmol. 2022 Jul 7;22:296. doi: 10.1186/s12886-022-02513-9 (PMC9260982; doi:10.1186/s12886-022-02513-9)
Supplement: Supplementary file 1 — Additional file 1: Case report form used by all participating centers in this retrospective study. [file 12886_2022_2513_MOESM1_ESM.pdf]

Case Report Form:

Occurrence and outcome of ocular firework injuries in Switzerland

Instruction for answering the Case Report Form:

1. **Only one choice → O**
2. **Multiple choice → ☐**

**Hospital_1**	**Consecutive Number** Abbreviation XXX	**Date** DD.MM.YYYY

Demographic Data

Age at time point of injury XX years
(In case of < 1 year: XX months)

Gender
O Male O Female

Date of injury DD.MM.YYYY O Unknown O Others: ___________
Date of first presentation DD.MM.YYYY

Role during firework
O Bystander O Operator O Unknown O others: please specify ________________

Injured eyes
O Right Eye O Left Eye O Both Eyes

Firework type
O Rocket
(Translation: German: Rakete; French: fusée; Italian: razzo pirotecnico)

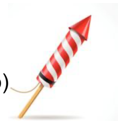

O Crackers
(Translation: German: Knallkörper/Böller; French: pétard; Italian: petardo)

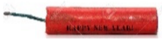

O Volcano fountain
(Translation: German: Fontäne/Vulkan; French: pétard; Italian: petardo)

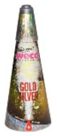

O Ground Spinners
(Translation: not available)

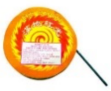

O Others: please specify ________________
O Unknown

Alcohol abuse at time point of firework injury
O Yes O No O Unknown O Others: please specify ________________

Drug abuse at time point of firework injury
O Yes O No O Unknown O Others: please specify ________________

Other parts of the body injured

☐ None
☐ Other parts of the head/face beside of eyes and lid
☐ Hands/fingers
☐ Arm
☐ Lower extremities
☐ Trunk
☐ Unknown
☐ Others: please specify _________________

Ophthalmological diagnosis <u>before</u> injury

☐ **Right Eye**	☐ **Left Eye**
☐ None<br>☐ Myopia<br>☐ Hyperopia<br>☐ Astigmatism<br>☐ Amblyopia<br>☐ Glaucoma<br>☐ Cataract<br>☐ Status post cataract surgery<br>☐ Status post refractive surgery<br>Type of intervention_______________<br>☐ Status post Lid surgery;<br>Type of intervention_______________<br>☐ Status post retinal surgery<br>Type of intervention_______________<br>☐ Status post glaucoma surgery<br>Type of intervention_______________<br>☐ Status post trauma<br>Please specify _________________<br>☐ Unknown<br>☐ Others: please specify<br>_________________<br>_________________	☐ None<br>☐ Myopia<br>☐ Hyperopia<br>☐ Astigmatism<br>☐ Amblyopia<br>☐ Glaucoma<br>☐ Cataract<br>☐ Status post cataract surgery<br>☐ Status post refractive surgery<br>Type of intervention_______________<br>☐ Status post Lid surgery;<br>Type of intervention_______________<br>☐ Status post retinal surgery<br>Type of intervention_______________<br>☐ Status post glaucoma surgery<br>Type of intervention_______________<br>☐ Status post trauma<br>Please specify _________________<br>☐ Unknown<br>☐ Others: please specify<br>_________________<br>_________________

Best corrected visual acuity after injury

Please provide information about both eyes in case of bilateral injury. In case of unilateral injury cross out the unaffected eye.

☐ Right Eye (if injured)	☐ Left Eye (if injured)
**I. At first presentation:**<br>O Unknown<br>O Non-light perception<br>O Light perception<br>O Hand movement<br>O Finger counting<br>O Decimal scale: X.XX (0.05 – 1.6)<br>O Others: please specify<br>__________________	**I. At first presentation:**<br>O Unknown<br>O Non-light perception<br>O Light perception<br>O Hand movement<br>O Finger counting<br>O Decimal scale: X.XX (0.05 – 1.6)<br>O Others: please specify<br>__________________
**II. One-year follow-up:**<br>O No one-year follow-up<br>O Unknown<br>O Non-light perception<br>O Light perception<br>O Hand movement<br>O Finger counting<br>O Decimal scale: X.XX (0.05 – 1.6)<br>O Others: please specify<br>__________________	**II. One-year follow-up:**<br>O No one-year follow-up<br>O Unknown<br>O Non-light perception<br>O Light perception<br>O Hand movement<br>O Finger counting<br>O Decimal scale: X.XX (0.05 – 1.6)<br>O Others: please specify<br>__________________
**III. At last follow-up**<br>DD.MM.YYYY<br>O No follow-up<br>O Unknown<br>O Non-light perception<br>O Light perception<br>O Hand movement<br>O Finger counting<br>O Decimal scale: X.XX (0.05 – 1.6)<br>O Others: please specify<br>__________________	**III. At last follow-up**<br>DD.MM.YYYY<br>O No follow-up<br>O Unknown<br>O Non-light perception<br>O Light perception<br>O Hand movement<br>O Finger counting<br>O Decimal scale: X.XX (0.05 – 1.6)<br>O Others: please specify<br>__________________

Intraocular pressure (IOP)

Please provide information about both eyes in case of bilateral injury. In case of unilateral injury cross out the unaffected eye.

☐ **Right Eye** (if injured)	☐ **Left Eye** (if injured)
**I. At first presentation:**<br>O XX mmHg<br>O Unknown<br>**Under IOP lowering therapy**<br>O Yes O No O Unknown<br>O Others: ________________<br><br>**St. p. Glaucoma surgery** MM.YYYY<br>O Yes O No O Unknown<br>*- if yes, specify* ______________________	**I. At first presentation:**<br>O XX mmHg<br>O Unknown<br>**Under IOP lowering therapy**<br>O Yes O No O Unknown<br>O Others: ________________<br><br>**St. p. Glaucoma surgery** MM.YYYY<br>O Yes O No O Unknown<br>*- if yes, specify* ______________________
**II. One-year follow-up:**<br>O XX mmHg<br>O No one-year follow-up<br>O Unknown<br>O Others:________________<br><br>**Under IOP lowering therapy**<br>O Yes O No O Unknown<br>O Others: ________________<br><br>**St. p. Glaucoma surgery** MM.YYYY<br>O Yes O No O Unknown<br>*- if yes, specify* ______________________	**II. One-year follow-up:**<br>O XX mmHg<br>O No one-year follow-up<br>O Unknown<br>O Others:________________<br><br>**Under IOP lowering therapy**<br>O Yes O No O Unknown<br>O Others: ________________<br><br>**St. p. Glaucoma surgery** MM.YYYY<br>O Yes O No O Unknown<br>*- if yes, specify* ______________________
**III. At last follow-up** DD.MM.YYYY**:**<br>O XX mmHg<br>O No follow-up<br>O Unknown<br>O Others:________________<br><br>**Under IOP lowering therapy**<br>O Yes O No O Unknown<br>O Others: ________________<br><br>**St. p. Glaucoma surgery** MM.YYYY<br>O Yes O No O Unknown<br>*- if yes, specify* ______________________	**III. At last follow-up** DD.MM.YYYY**:**<br>O XX mmHg<br>O No follow-up<br>O Unknown<br>O Others:________________<br><br>**Under IOP lowering therapy**<br>O Yes O No O Unknown<br>O Others: ________________<br><br>**St. p. Glaucoma surgery** MM.YYYY<br>O Yes O No O Unknown<br>*- if yes, specify* ______________________

Maximal Intraocular Pressure (IOP)

Please provide information about both eyes in case of bilateral injury. In case of unilateral injury cross out the unaffected eye.

☐ **Right Eye** (if injured)	☐ **Left Eye** (if injured)
**I. In the first week:**<br>O Max. XX mmHg on DD.MM.YYYY<br>O No follow-up<br>O Unknown<br>O Others:_________________	**I. In the first week:**<br>O Max. XX mmHg on DD.MM.YYYY<br>O No follow-up<br>O Unknown<br>O Others:_________________
**II. In the first month:**<br>O Max. XX mmHg on DD.MM.YYYY<br>O No follow-up<br>O Unknown<br>O Others:_________________	**II. In the first month:**<br>O Max. XX mmHg on DD.MM.YYYY<br>O No follow-up<br>O Unknown<br>O Others:_________________
**III. In the first year:**<br>O Max. XX mmHg on DD.MM.YYYY<br>O No follow-up<br>O Unknown<br>O Others:_________________	**III. In the first year:**<br>O Max. XX mmHg on DD.MM.YYYY<br>O No follow-up<br>O Unknown<br>O Others:_________________

Findings at first presentation

Please provide information about both eyes in case of bilateral injury. In case of unilateral injury cross out the unaffected eye.

☐ **Right Eye** (if injured)	☐ **Left Eye** (if injured)
**Anterior segment findings**<br><br>**I. Lid and lacrimal system**<br>☐ None<br>☐ Eyelid laceration<br>☐ Traumatic ptosis<br>☐ Lid burn<br>☐ Charred eyelashes<br>☐ Canalicular laceration<br>☐ Others_____________<br><br>**II. Conjunctiva and sklera**<br>☐ None<br>☐ Hyposphagma<br>☐ Chemosis<br>☐ Conjunctival foreign body<br>☐ Ash<br>☐ Solid particles<br>☐ Conjunctival burn<br>☐ Conjunctival laceration, if yes<br>O XX mm x XX mm<br>O unknown<br>O others: ___________<br>☐ superior<br>☐ inferior<br>☐ temporal<br>☐ nasal<br>☐ Scleral laceration; if yes:<br>O > 3.5mm away from the limbus<br>O < 3.5mm away from the limbus<br>O Others: ____________<br>☐ Corneoscleral laceration<br>☐ Others_____________<br><br>**III. Cornea**<br>☐ None<br>☐ Corneal foreign body<br>☐ central<br>☐ peripheral<br>☐ others: ___________<br>☐ Corneal burn	**Anterior segment findings**<br><br>**I. Lid and lacrimal system**<br>☐ None<br>☐ Eyelid laceration<br>☐ Traumatic ptosis<br>☐ Lid burn<br>☐ Charred eyelashes<br>☐ Canalicular laceration<br>☐ Others_____________<br><br>**II. Conjunctiva and sklera**<br>☐ None<br>☐ Hyposphagma<br>☐ Chemosis<br>☐ Conjunctival foreign body<br>☐ Ash<br>☐ Solid particles<br>☐ Conjunctival burn<br>☐ Conjunctival laceration, if yes<br>O XX mm x XX mm<br>O unknown<br>O others: ___________<br>☐ superior<br>☐ inferior<br>☐ temporal<br>☐ nasal<br>☐ Scleral laceration; if yes:<br>O > 3.5mm away from the limbus<br>O < 3.5mm away from the limbus<br>O Others: ____________<br>☐ Corneoscleral laceration<br>☐ Others_____________<br><br>**III. Cornea**<br>☐ None<br>☐ Corneal foreign body<br>☐ central<br>☐ peripheral<br>☐ others: ___________<br>☐ Corneal burn

☐ central<br>☐ peripheral<br>☐ others: ___________<br>☐ Corneal epithelial defect<br>☐ central<br>☐ peripheral<br>☐ others: ___________<br>☐ Corneal perforation<br>☐ central<br>☐ peripheral<br>O tissue defect: XX mm x XX mm<br>O cut: XX mm<br>O unknown<br>O others: ____________<br>☐ Corneal laceration; if yes:<br>☐ central<br>☐ peripheral<br>O area: XX mm x XX mm<br>O cut: XX mm<br>O unknown<br>O others: ____________<br>☐ Others_____________<br><br>**IV. Anterior chamber, iris, lens, pupil**<br>☐ None<br>☐ Iridodialysis<br>☐ Aniridia<br>☐ Traumatic mydriasis<br>☐ Intracameral foreign body<br>☐ Traumatic iritis<br>☐ 0.5+ ☐ 1+ ☐ 2+ ☐ 3+<br>☐ Hyphemia, if yes:<br>O Height in mm XX<br>O Total Hyphemia<br>O Others ____________<br>☐ RAPD (relative afferent pupillary defect)<br>☐ Angle recession<br>☐ Lens subluxation<br>☐ Lens dislocation<br>☐ Traumatic cataract<br>☐ Ciliary body detachment<br>☐ Others _____________	☐ central<br>☐ peripheral<br>☐ others: ___________<br>☐ Corneal epithelial defect<br>☐ central<br>☐ peripheral<br>☐ others: ___________<br>☐ Corneal perforation<br>☐ central<br>☐ peripheral<br>O tissue defect: XX mm x XX mm<br>O cut: XX mm<br>O unknown<br>O others: ____________<br>☐ Corneal laceration; if yes:<br>☐ central<br>☐ peripheral<br>O area: XX mm x XX mm<br>O cut: XX mm<br>O unknown<br>O others: ____________<br>☐ Others_____________<br><br>**IV. Anterior chamber, iris, lens, pupil**<br>☐ None<br>☐ Iridodialysis<br>☐ Aniridia<br>☐ Traumatic mydriasis<br>☐ Intracameral foreign body<br>☐ Traumatic iritis<br>☐ 0.5+ ☐ 1+ ☐ 2+ ☐ 3+<br>☐ Hyphemia, if yes:<br>O Height in mm XX<br>O Total Hyphemia<br>O Others ____________<br>☐ RAPD (relative afferent pupillary defect)<br>☐ Angle recession<br>☐ Lens subluxation<br>☐ Lens dislocation<br>☐ Traumatic cataract<br>☐ Ciliary body detachment<br>☐ Others: _____________
**Posterior segment findings**<br>☐ None<br>☐ Vitreous hemorrhage	**Posterior segment findings**<br>☐ None<br>☐ Vitreous hemorrhage

☐ Retinal hemorrhage<br>☐ Retinal contusion/ Berlin's edema<br>☐ Traumatic optic neuropathy<br>☐ Retinal detachment<br>☐ Choroidal detachment<br>☐ Choroidal rupture<br>☐ Traumatic retinal foramen<br>☐ Giant retinal tear<br>☐ Traumatic macular hole<br>☐ Intravitreal foreign body<br>☐ Retinal foreign body<br>☐ Endophthalmitis<br>☐ Others _____________	☐ Retinal hemorrhage<br>☐ Retinal contusion/ Berlin's edema<br>☐ Traumatic optic neuropathy<br>☐ Retinal detachment<br>☐ Choroidal detachment<br>☐ Choroidal rupture<br>☐ Traumatic retinal foramen<br>☐ Giant retinal tear<br>☐ Traumatic macular hole<br>☐ Intravitreal foreign body<br>☐ Retinal foreign body<br>☐ Endophthalmitis<br>☐ Others _____________
**General findings**<br>☐ None<br>☐ Globe rupture<br>☐ Traumatic ophthalmoplegia<br>☐ Orbital fracture<br>☐ Traumatic retrobulbar hemorrhage<br>☐ Others _____________	**General findings**<br>☐ None<br>☐ Globe rupture<br>☐ Traumatic ophthalmoplegia<br>☐ Orbital fracture<br>☐ Traumatic retrobulbar hemorrhage<br>☐ None<br>☐ Others _____________

Primary surgical management

O Yes O No O Unknown **If yes**, please fill out the table:

Please provide information about both eyes in case of bilateral injury. In case of unilateral injury cross out the unaffected eye.

☐ **Right Eye** (if injured)	☐ **Left Eye** (if injured)
☐ Globe exploration<br>☐ Canthotomy<br>☐ Eyelid suture<br>☐ Conjunctival suture repair<br>☐ Extraction of conjunctival foreign bodies<br>☐ Scleral suture repair<br>☐ Symblepharon ring<br>☐ Abrasio<br>☐ Corneal suture repair<br>☐ Corneoscleral suture repair<br>☐ Extraction of corneal foreign bodies<br>☐ Penetrating keratoplasty à chaud<br>☐ Amniotic membrane graft<br>☐ Iris repair<br>☐ Lensectomy without intraocular lens insertion<br>☐ Lensectomy with intraocular lens insertion<br>☐ Extraction of intraocular foreign bodies<br>☐ Anterior chamber washout<br>☐ Laser coagulation of a retinal foramen<br>☐ Cryotherapy of a retinal foramen<br>☐ Vitrectomy<br>O Gas<br>O Silicon Oil<br>O BSS<br>☐ Cryotherapy<br>☐ Endolaser<br>☐ Retinectomy<br>☐ Enucleation/Evisceration<br>☐ Others: please specify<br>________________________<br>________________________	☐ Globe exploration<br>☐ Canthotomy<br>☐ Eyelid suture<br>☐ Conjunctival suture repair<br>☐ Extraction of conjunctival foreign bodies<br>☐ Scleral suture repair<br>☐ Symblepharon ring<br>☐ Abrasio<br>☐ Corneal suture repair<br>☐ Corneoscleral suture repair<br>☐ Extraction of corneal foreign bodies<br>☐ Penetrating keratoplasty à chaud<br>☐ Amniotic membrane graft<br>☐ Iris repair<br>☐ Lensectomy without intraocular lens insertion<br>☐ Lensectomy with intraocular lens insertion<br>☐ Extraction of intraocular foreign bodies<br>☐ Anterior chamber washout<br>☐ Laser coagulation of a retinal foramen<br>☐ Cryotherapy of a retinal foramen<br>☐ Vitrectomy<br>O Gas<br>O Silicon Oil<br>O BSS<br>☐ Cryotherapy<br>☐ Endolaser<br>☐ Retinectomy<br>☐ Enucleation/Evisceration<br>☐ Others: please specify<br>________________________<br>________________________

Primary non-surgical (systemic and topical) management

O Yes O No O Unknown **If yes**, please fill out the table:

Please provide information about both eyes in case of bilateral injury. In case of unilateral injury cross out the unaffected eye.

☐ **Right Eye** (if injured)	☐ **Left Eye** (if injured)
**Topical**<br>☐ Irrigation<br>☐ Artificial tears<br>☐ Intravitreal antibiotics<br>☐ Topical antibiotics<br>☐ Topical corticosteroids<br>☐ Topical NSAR<br>☐ Cycloplegics<br>☐ IOP lowering agents, topical<br>☐ Bandage lens<br>**Systemic**<br>☐ Systemic antibiotics (oral and intravenous)<br>☐ Systemic NSAR<br>☐ Systemic corticosteroids<br>☐ IOP lowering agents, oral<br>☐ Tetanus Prophylaxis<br>☐ Others: please specify<br>___________________	**Topical**<br>☐ Irrigation<br>☐ Artificial tears<br>☐ Intravitreal antibiotics<br>☐ Topical antibiotics<br>☐ Topical corticosteroids<br>☐ Topical NSAR<br>☐ Cycloplegics<br>☐ IOP lowering agents, topical<br>☐ Bandage lens<br>**Systemic**<br>☐ Systemic antibiotics (oral and intravenous)<br>☐ Systemic NSAR<br>☐ Systemic corticosteroids<br>☐ IOP lowering agents, oral<br>☐ Tetanus Prophylaxis<br>☐ Others: please specify<br>___________________

Hospitalization:

Hospitalization directly after the trauma and first presentation

O Yes O No

- **If yes**: hospitalization for XXX days

Secondary hospitalization at later time points (e.g. due to complications or surgeries during follow-up)

O Yes O No

- **If yes**: hospitalization for a total of XXX days

Duration of sick leave:

☐ 100% over XXX day(s) or XXX month(s) or XXX year(s)
☐ 50% over XXX day(s) or XXX month(s) or XXX year(s)
☐ Other: ___ % over XXX day(s) or XXX month(s) or XXX year(s)
☐ Other: ___ % over XXX day(s) or XXX month(s) or XXX year(s)
☐ Invalidity/disability since DD.MM.YYYY; XXX %
(German: Invalidenversicherung; French: l'assurance-invalidité; Italian: sull'assicurazione invalidità)
☐ Unknown
☐ None
☐ Others: please specify ___________________

Number of ophthalmological consultations: XXX

Secondary Complications

O Yes O No O Unknown **If yes**, please fill out the table:

Please provide information about both eyes in case of bilateral injury. In case of unilateral injury cross out the unaffected eye.

☐ **Right Eye** (if injured)	☐ **Left Eye** (if injured)
☐ Entropion<br>☐ Ectropion<br>☐ Trichiasis<br>☐ Symblepharon<br>☐ Corneal scar<br>☐ central<br>☐ peripheral<br>☐ total<br>☐ unknown<br>☐ others: ____________<br>O XX mm x XX mm<br>☐ Corneal decompensation<br>☐ Corneal perforation<br>☐ central<br>☐ peripheral<br>O tissue defect: XX mm x XX mm<br>O cut: XX mm<br>O unknown<br>O others: ____________<br>☐ Keratoconjunctivitis sicca<br>☐ Limbal Stem Cell Deficiency<br>☐ Cataract (traumatic, postoperative etc.)<br>☐ Steroid induced ocular hypertension<br>☐ Secondary glaucoma<br>O steroid induced glaucoma<br>O unknown etiology<br>O others: ____________<br>☐ Uveitis<br>☐ anterior<br>☐ intermedia<br>☐ posterior<br>☐ Phtisis bulbi<br>☐ Macular scarring<br>☐ Retinal detachment, Macula on<br>☐ Retinal detachment, Macula off<br>☐ Retinal scar<br>☐ Retinal Foramina<br>☐ Macular Foramina<br>☐ Choroidal neovascularization	☐ Entropion<br>☐ Ectropion<br>☐ Trichiasis<br>☐ Symblepharon<br>☐ Corneal scar<br>☐ central<br>☐ peripheral<br>☐ total<br>☐ unknown<br>☐ others: ____________<br>O XX mm x XX mm<br>☐ Corneal decompensation<br>☐ Corneal perforation<br>☐ central<br>☐ peripheral<br>O tissue defect: XX mm x XX mm<br>O cut: XX mm<br>O unknown<br>O others: ____________<br>☐ Keratoconjunctivitis sicca<br>☐ Limbal Stem Cell Deficiency<br>☐ Cataract (traumatic, postoperative etc.)<br>☐ Steroid induced ocular hypertension<br>☐ Secondary glaucoma<br>O steroid induced glaucoma<br>O unknown etiology<br>O others: ____________<br>☐ Uveitis<br>☐ anterior<br>☐ intermedia<br>☐ posterior<br>☐ Phtisis bulbi<br>☐ Macular scarring<br>☐ Retinal detachment, Macula on<br>☐ Retinal detachment, Macula off<br>☐ Retinal scar<br>☐ Retinal Foramina<br>☐ Macular Foramina<br>☐ Choroidal neovascularization

☐ Traumatic optic neuropathy<br>☐ Sympathetic ophthalmia<br>☐ Others: please specify<br>______________________<br>______________________	☐ Traumatic optic neuropathy<br>☐ Sympathetic ophthalmia<br>☐ Others: please specify<br>______________________<br>______________________

Secondary non-surgical (systemic and topical) management

O Yes O No O Unknown **If yes**, please fill out the table:

Please provide information about both eyes in case of bilateral injury. In case of unilateral injury cross out the unaffected eye.

☐ **Right Eye** (if injured)	☐ **Left Eye** (if injured)
**Topical**<br>☐ Artificial tears<br>☐ Autologous serum eye drops<br>☐ Intravitreal antibiotics<br>☐ Topical antibiotics<br>☐ Topical corticosteroids<br>☐ Topical NSAR<br>☐ Topical Immunosuppressants (e.g. Ikervis)<br>☐ Cycloplegics<br>☐ IOP lowering agents, topical<br>☐ Bandage lens<br>☐ Anti-VEGF<br>☐ Intravitreal corticosteroids<br>☐ Others: please specify<br>______________________<br>______________________<br>**Systemic**<br>☐ Systemic NSAR<br>☐ Systemic antibiotics (oral and intravenous)<br>☐ Systemic corticosteroids<br>☐ Systemic Immunosuppressants<br>☐ IOP lowering agents, oral<br>☐ Others: please specify<br>______________________<br>______________________	**Topical**<br>☐ Artificial tears<br>☐ Autologous serum eye drops<br>☐ Intravitreal antibiotics<br>☐ Topical antibiotics<br>☐ Topical corticosteroids<br>☐ Topical NSAR<br>☐ Topical Immunosuppressants (e.g. Ikervis)<br>☐ Cycloplegics<br>☐ IOP lowering agents, topical<br>☐ Bandage lens<br>☐ Anti-VEGF<br>☐ Intravitreal corticosteroids<br>☐ Others: please specify<br>______________________<br>______________________<br>**Systemic**<br>☐ Systemic NSAR<br>☐ Systemic antibiotics (oral and intravenous)<br>☐ Systemic corticosteroids<br>☐ Systemic Immunosuppressants<br>☐ IOP lowering agents, oral<br>☐ Others: please specify<br>______________________<br>______________________

Secondary surgical management

O Yes O No O Unknown **If yes**, please fill out the table:
In case of consecutive surgeries, please fill in the dates in the sub item.
Please provide information about both eyes in case of bilateral injury. In case of unilateral injury cross out the unaffected eye.

☐ **Right Eye** (if injured)	☐ **Left Eye** (if injured)
☐ **Repair of eyelid**, MM. YYYY<br>2. MM. YYYY<br>3. MM. YYYY<br>☐ **Electrical depilation**, MM. YYYY<br>2. MM. YYYY<br>3. MM. YYYY<br>☐ Symblepharon ring MM. YYYY<br>2. MM. YYYY<br>3. MM. YYYY<br>☐ **Symblepharon separation**, MM. YYYY<br>2. MM. YYYY<br>3. MM. YYYY<br>☐ **Conjunctival flap**, MM. YYYY<br>2. MM. YYYY<br>3. MM. YYYY<br>☐ **Corneal suture repair**, MM. YYYY<br>2. MM. YYYY<br>3. MM. YYYY<br>☐ **Scleral suture repair**, MM. YYYY<br>2. MM. YYYY<br>3. MM. YYYY<br>☐ **Corneoscleral suture repair**, MM. YYYY<br>2. MM. YYYY<br>3. MM. YYYY<br>☐ **Corneal transplantation**<br>☐ Penetrating keratoplasty<br>☐ DALK (Deep Anterior Lamellar Keratoplasty)<br>☐ DMEK (Descemet Membrane Endothelial Keratoplasty)<br>☐ DSAEK (Descemet's Stripping Endothelial Keratoplasty)<br>1. MM. YYYY<br>2. MM. YYYY<br>3. MM. YYYY<br>☐ **Limbal stem cell transplant**, MM. YYYY<br>2. MM. YYYY<br>3. MM. YYYY<br>☐ **Amniotic membrane graft**,MM. YYYY<br>2. MM. YYYY<br>3. MM. YYYY<br>☐ **Phototherapeutic keratectomy**	☐ **Repair of eyelid**, DD. MM. YYYY<br>2. MM. YYYY<br>3. MM. YYYY<br>☐ **Electrical depilation**, DD. MM. YYYY<br>2. MM. YYYY<br>3. MM. YYYY<br>☐ Symblepharon ring MM. YYYY<br>2. MM. YYYY<br>3. MM. YYYY<br>☐ **Symblepharon separation**, DD. MM. YYYY<br>2. MM. YYYY<br>3. MM. YYYY<br>☐ **Conjunctival flap**, DD. MM. YYYY<br>2. MM. YYYY<br>3. MM. YYYY<br>☐ **Corneal suture repair**, MM. YYYY<br>2. MM. YYYY<br>3. MM. YYYY<br>☐ **Scleral suture repair**, MM. YYYY<br>2. MM. YYYY<br>3. MM. YYYY<br>☐ **Corneoscleral suture repair**, MM. YYYY<br>2. MM. YYYY<br>3. MM. YYYY<br>☐ **Corneal transplantation**<br>☐ Penetrating keratoplasty<br>☐ DALK (Deep Anterior Lamellar Keratoplasty)<br>☐ DMEK (Descemet Membrane Endothelial Keratoplasty)<br>☐ DSAEK (Descemet's Stripping Endothelial Keratoplasty)<br>1. MM. YYYY<br>2. MM. YYYY<br>3. MM. YYYY<br>☐ **Limbal stem cell transplant**, MM. YYYY<br>2. MM. YYYY<br>3. MM. YYYY<br>☐ **Amniotic membrane graft**, MM. YYYY<br>2. MM. YYYY<br>3. MM. YYYY<br>☐ **Phototherapeutic keratectomy**

1. MM. YYYY<br>2. MM. YYYY<br>3. MM. YYYY<br>☐ **Keratoprostehsis,** MM. YYYY<br>☐ **Iris repair**, MM. YYYY<br>2. MM. YYYY<br>3. MM. YYYY<br>☐ **Peripheral iridectomy**, MM. YYYY<br>2. MM. YYYY<br>3. MM. YYYY<br>☐ **Laser peripheral iridotomy,** MM. YYYY<br>2. MM. YYYY<br>3. MM. YYYY<br>☐ **Lensectomy with intraocular lens insertion,** MM. YYYY<br>☐ **Secondary intraocular lens insertion**<br>1. MM. YYYY<br>2. MM. YYYY<br>3. MM. YYYY<br>☐ **Laser coagulation of a retinal foramen**<br>1. MM. YYYY<br>2. MM. YYYY<br>3. MM. YYYY<br>☐ **Cryotherapy of a retinal foramen**<br>1. MM. YYYY<br>2. MM. YYYY<br>3. MM. YYYY<br>☐ **Vitrectomy**, MM. YYYY<br>2. MM. YYYY<br>3. MM. YYYY<br>☐ Gas<br>☐ Silicon Oil<br>☐ BSS<br>☐ Cryotherapy<br>☐ Endolaser<br>☐ Retinectomy<br>☐ **Glaucoma surgery**<br>☐ Trabeculectomy, MM. YYYY<br>☐ Deep sclerectomy, MM. YYYY<br>☐ Stents/Tubes, MM. YYYY<br>☐ Others : ________________________, MM. YYYY<br>☐ **Eviszeration/Enucleation** MM. YYYY<br>☐ **Others:**<br>________________________,MM. YYYY<br>________________________,MM. YYYY	1. MM. YYYY<br>2. MM. YYYY<br>3. . MM. YYYY<br>☐ **Keratoprostehsis,** MM. YYYY<br>☐ **Iris repair**, MM. YYYY<br>2. . MM. YYYY<br>3. MM. YYYY<br>☐ **Peripheral iridectomy**, MM. YYYY<br>2. MM. YYYY<br>3. . MM. YYYY<br>☐ **Laser peripheral iridotomy,** MM. YYYY<br>2. MM. YYYY<br>3. . MM. YYYY<br>☐ **Lensectomy with intraocular lens insertion,** MM. YYYY<br>☐ **Secondary intraocular lens insertion**<br>1. MM. YYYY<br>2. MM. YYYY<br>3. MM. YYYY<br>☐ **Laser coagulation of a retinal foramen**<br>1. MM. YYYY<br>2. MM. YYYY<br>3. MM. YYYY<br>☐ **Cryotherapy of a retinal foramen**<br>1. MM. YYYY<br>2. MM. YYYY<br>3. MM. YYYY<br>☐ **Vitrectomy**, MM. YYYY<br>2. MM. YYYY<br>3. MM. YYYY<br>☐ Gas<br>☐ Silicon Oil<br>☐ BSS<br>☐ Cryotherapy<br>☐ Endolaser<br>☐ Retinectomy<br>☐ **Glaucoma surgery**<br>☐ Trabeculectomy, MM. YYYY<br>☐ Deep sclerectomy, MM. YYYY<br>☐ Stents/Tubes, MM. YYYY<br>☐ Others : ________________________, MM. YYYY<br>☐ **Eviszeration/Enucleation** MM. YYYY<br>☐ **Others:**<br>________________________, MM. YYYY<br>________________________,MM. YYYY

____________________________, MM. YYYY	____________________________, MM. YYYY
____________________________, MM. YYYY	____________________________, MM. YYYY
____________________________, MM. YYYY	____________________________, MM. YYYY
____________________________, MM. YYYY	____________________________, MM. YYYY
____________________________, MM. YYYY	____________________________, MM. YYYY
____________________________, MM. YYYY	____________________________, MM. YYYY
____________________________, MM. YYYY	____________________________, MM. YYYY
____________________________, MM. YYYY	____________________________, MM. YYYY
____________________________, MM. YYYY	____________________________, MM. YYYY
____________________________, MM. YYYY	____________________________, MM. YYYY
____________________________, MM. YYYY	____________________________, MM. YYYY
____________________________, MM. YYYY	____________________________, MM. YYYY
